# Anthocyanin analyses of *Vaccinium* fruit dietary supplements

**DOI:** 10.1002/fsn3.339

**Published:** 2016-01-20

**Authors:** Jungmin Lee

**Affiliations:** ^1^ United States Department of AgricultureAgricultural Research Service (ARS)Horticultural Crops Research Unit (HCRU) WorksiteParmaIdaho83660

**Keywords:** Adulteration, anthocyanin, dietary supplements, quality, *Vaccinium*

## Abstract

*Vaccinium* fruit ingredients within dietary supplements were identified by comparisons with anthocyanin analyses of known *Vaccinium* profiles (demonstration of anthocyanin fingerprinting). Available *Vaccinium* supplements were purchased and analyzed, their anthocyanin profiles (based on high‐performance liquid chromatography [HPLC] separation) indicated if products' fruit origin listings were authentic. Over 30% of the *Vaccinium* fruit (cranberry, lingonberry, bilberry, and blueberry; 14 of 45) products available as dietary supplements did not contain the fruit listed as ingredients. Six supplements contained no anthocyanins. Five others had contents differing from labeled fruit (e.g., bilberry capsules containing Andean blueberry fruit). Of the samples that did contain the specified fruit (*n* = 27), anthocyanin content ranged from 0.04 to 14.37 mg per capsule, tablet, or teaspoon (5 g). Approaches to utilizing anthocyanins in assessment of sample authenticity, and a discussion of the challenges with anthocyanin profiles in quality control are both presented.

## Introduction

The fruit‐based dietary supplement business is thriving, though their economic value may be difficult to ascertain. In 2009 the Natural Products Foundation (Washington, DC) said the entire dietary supplement industry made an estimated $61 billion in the United States, and in 2011 the industry made $151 billion globally (Mondello 2013). It may not be surprising with such sales that adverse events reported to U.S. FDA (Food and Drug Administration) due to dietary supplements have been increasing, more than doubling from approximately 1000 events in 2010 to over 2800 in 2012. Since people purchase and consume dietary supplements to improve overall health or to fill nutrition gaps in their daily diet (Dickinson et al. 2014), consumers might find it disturbing to learn that what they consume for health benefits does not always contain, or has only small amounts of, what is on the label. Industry stakeholders upholding high‐quality dietary supplements are requesting a quality assurance technique to authenticate ingredients and products (Anonymous, pers. comm.).

Marketplace food, beverage, and dietary supplement adulteration is nothing new. In 1906, when the FDA was created, one of its chief responsibilities was the investigation of food product adulteration. Fruit product adulteration was reported in the scientific literature as early as 1884 when red small fruit jams were found adulterated with cheaper apple pulp (Adams 1884). Over a century later, sourcing cheaper unlisted fruit for ingredients, or outright imitation of more exclusive fruit products are still common occurrences (Adams 1884; Wrolstad et al. 1982; Lee 2015). In 2015, New York Attorney General (E. Schneiderman) made media headlines by announcing the findings of a dietary supplement adulteration investigation. Based on DNA test results, his office issued cease‐and‐desist letters to major dietary supplement retailers for the products allegedly lacking listed ingredients, or that contained unlabeled ingredients. *Vaccinium* fruit‐based dietary supplements were not sampled or tested on this occasion.

Recent efforts have been made to improve the quality of dietary supplements offered to consumers in the United States. The National Institutes of Health–Office of Dietary Supplements (NIH‐ODS) and AOAC (Association of Analytical Communities) International have set out to collaboratively establish analytical method standards that promote effective quality control of *Vaccinium* fruit‐based ingredients in dietary supplements. Their goal is the establishment of a standard method, officially approved by AOAC International, applicable for quality assurance evaluations of anthocyanin‐containing dietary supplements (one of the 25 standard method performance requirements for dietary supplements ingredients). A systematic database of anthocyanin profiles is also needed. Its availability would allow manufacturers to make comparisons, confirm ingredient authenticity, or reveal undeclared content in a source material. Some phenolic databases are already available, including Phenol‐Explorer (Rothwell et al. 2012; http://www.phenol-exploerer.eu) and the USDA's phytonutrient database (USDA [United States Department of Agriculture] 2015); although the USDA database currently only has anthocyanidin data (no anthocyanins yet). Even with thorough and reliable anthocyanin datasets, the ease in which one species of small fruit can be distinguished from another (or even cultivars of a single species) through their profiles varies a great deal (Lee and Finn 2007, 2012; Lee 2015).

As U.S. dietary supplement industry have yet to define standards for minimum active component concentration or maximum daily consumption, the currently available products represent a vast range of quality (Lee 2010, 2013, 2014). For example, previous work on U.S. black raspberry (*Rubus occidentalis* L.) dietary supplements confirmed that 36% of investigated samples did not contain any black raspberry fruit (Lee 2014). Many products that claimed to have been sourced from Korean black raspberry (*R. coreanus* Miq.) fruit were found to contain *R. occidentalis* L., or in one extreme case only the extracts of black carrots (Lee 2015).

The objective of this work was to demonstrate if anthocyanin profiles can still be utilized to authenticate (qualitative anthocyanin fingerprinting) marketplace *Vaccinium* species fruit dietary supplements.

## Materials and Methods

### Supplement samples and extraction

**Figure 1:**
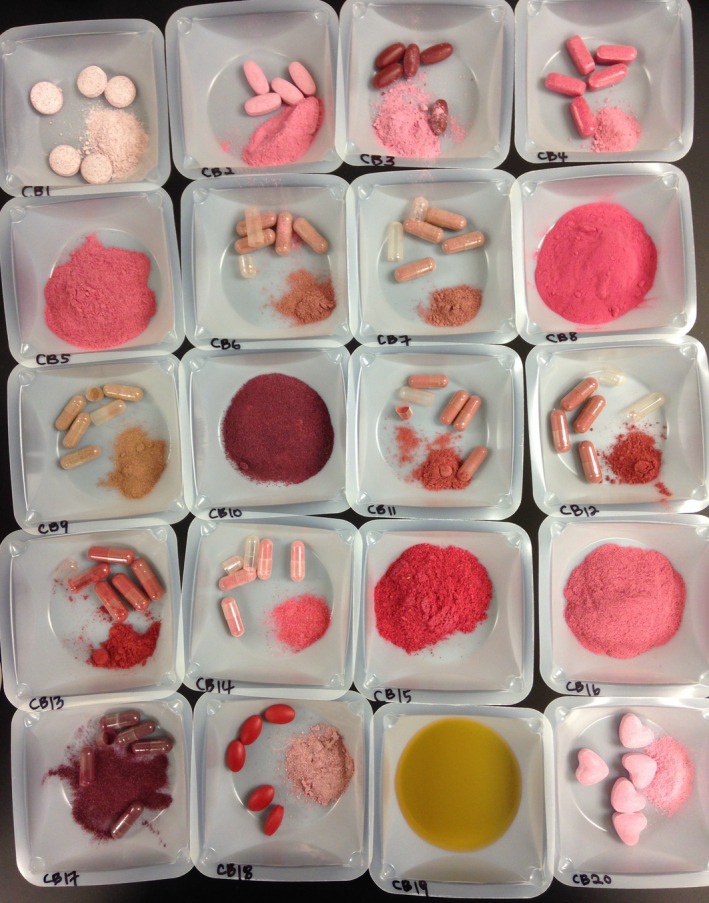
Cranberry dietary supplement bottle contents presented here as an example. Tablets were powdered and capsule contents were emptied and presented next to the original form.

All available dietary supplements (Table 1), including herbal supplements, labeled to contain either cranberry (coded CB1‐CB20; *Vaccinium macrocarpon* Ait.; also known as American cranberry; see Fig. 1), lingonberry (LB1‐LB2; *V. vitis‐idaea* L.), bilberry (BL1‐BL15; typically *V. myrtillus* L.; also known as European bilberry), or blueberry (BB1‐BB8; typically *V. corymbosum* L.; also known as highbush blueberry) were purchased from Amazon Marketplace (Seattle, WA) and locally (Boise, Nampa, and Caldwell, ID) in June 2014. All products were represented with a code (e.g., CB1), since publishing the company name was not an objective. The purchased products represented 31 separate companies, and all were analyzed prior their expiration, or best use by, date. These *Vaccinium* samples were packaged as loose powders, capsules, tablets, liquid extracts, and dried fruit forms (*n* = 45; Table 1). Soft‐gel capsule supplements were excluded from this study as they also contain nonfruit ingredients (soybean oil, rice bran oil, beeswax, etc.), requiring different extraction procedures from the other samples. The shells of encapsulated products were removed and contents pooled prior to extractions, and weights were recorded (both capsules and powder) for later calculation of anthocyanin per capsule. An IKA Tube Mill control (IKA Works, Inc., Wilmington, NC) and 40 mL disposable grinding chambers were used to grind tablet and dried fruit samples into powder. After powdering they were extracted with water, following without deviation as described in Lee (2014). Extractions were conducted in duplicates.

**Table 1:** Sample codes and brief summary of label information of cranberry (CB; *n* = 20), lingonberry (LB; *n* = 2), bilberry (BL; *n* = 15), and blueberry (BB; *n* = 8) dietary supplements

Sample code	Form	Relevant ingredient listings and information from product label
CB1	Tablet	Cranberry concentrate, xylitol, cellulose gum, PVP, natural cranberry flavor, silica, stearic acid, malic acid, magnesium stearate, citric acid, modified food starch, malodextrin, tartaric acid, guar gum, sunflower lecithin, and no milk, egg, fish, crustacean shellfish, tree nuts, peanuts, soy nuts, yeast, artificial colors, flavors, or preservatives.
CB2	Tablet	Cranberry (berry), cellulose, modified cellulose, silica, modified cellulose gum, and stearic acid.
CB3	Tablet	Cranberry, dicalcium phosphate, microcrystalline cellulose, hypromellose, magnesium hydroxide, croscarmellose sodium, silicon dioxide, magnesium stearate, stearic acid, polyethylene glycol, carmine color, dextrin, caramel color, dextrose, lecithin, sodium carboxymethyl cellulose, sodium citrate, and contain soy.
CB4	Tablet	Cranberry concentrate, dicalcium phosphate hyroxypropyl methylcellulose, stearic acid, microcrystalline cellulose, magnesium stearate, silica, juniper berry (*Juniperus communis*), parsley (*Petroselinum crispum*), red clover (*Trifolium pratense*), uva‐ursi (*Arctostaphylos uva‐ursi*), and pharmaceutical glaze.
CB5	Loose powder	Cranberry 36:1 extract powder, organic, no fillers, and no chemicals or preservatives.
CB6	Capsule	Cranberry, *Vaccinium macrocarpon* extract, gelatin capsule (gelatin, purified water), rice powder, silica, and magnesium stearate (vegetable grade).
CB7	Capsule	Cranberry (*V. macrocarpon*) concentrate (36:1) fruit, gelatin capsule (gelatin, purified water), rice powder, silica, magnesium stearate (vegetable grade).
CB8	Loose powder	Cranberry juice powder, freeze dried organic *V. macrocarpon*, country of origin – USA.
CB9	Capsule	Cranberry fruit, dandelion leaf, marshmallow root, cleavers (stem, leaf, fruit, and flower), corn silk, goldenseal root, gelatin (capsule), magnesium stearate, and silica.
CB10	Loose powder	Dried cranberries, no sugar added, and gluten, dairy, and allergen free.
CB11	Capsule	Cranberry juice powder, *V. macrocarpon* fruit, gelatin, silica, and no artificial color.
CB12	Capsule	Cranberry powder, *V. macrocarpon* fruit, and gelatin.
CB13	Capsule	Cranberry (*V. macrocarpon*), gelatin (capsule), and magnesium stearate (vegetable source).
CB14	Capsule	Cranberry fruit, gelatin, and silica.
CB15	Loose powder	Powdered freeze dried cranberry, *V. macrocarpon*, nonirradiated.
CB16	Loose powder	Cranberry powder and silica.
CB17	Capsule	Cranberry juice extract, gelatin, microcrystalline cellulose, magnesium stearate, and silicon dioxide
CB18	Tablet	Natural cranberry powder, dicalcium phosphate, microcrystalline cellulose, stearic acid, povidone, coating (hypromellose, polyethylene glycol, triacetin, and colors [titanium dioxide, FD&C Red #40, and FD&C Blue #2]), hypromellose, magnesium stearate, and silicon dioxide, and contains egg.
CB19	Liquid extract	Trade name (proprietary blend) – cranberry concentrate, filtered water, FOS (fructooligosaccharides), bromelain, glycerin, acesulfame‐K, sucralose, phosphoric acid, sodium benzoate (preservative) and potassium sorbate (preservative), and lactose, gluten, and sugar free.
CB20	Tablet	Cranberry with vitamin C, natural cranberry/strawberry flavor, non GMO, gluten free, fructose, stearic acid, natural strawberry flavor, magnesium stearate silica, and beet juice.
LB1	Capsule	Lingonberry, modified cellulose (vegetarian capsule), microcrystalline cellulose, magnesium stearate, and silica.
LB2	Capsule	Lingonberry fruit, gelatin, rice flour, and silica.
BL1	Capsule	Bilberry fruit extract (*V. myrtillus*) cellulose gel, gelatin (nonbovine), stearic acid, and water
BL2	Capsule	Bilberry fruit extract (*V. myrtillus*), dicalcium phosphate, cellulose, and vegetable cellulose capsule.
BL3	Capsule	European bilberry (*V. myrtillus* L.) extract (fruit), rice flour, vegetable cellulose (capsule), and l‐leucine
BL4	Tablet	Bilberry fruit (*V. myrtillus*), dibasic calcium phosphate, stearic acid, microcrystalline cellulose, modified cellulose gum, and silica.
BL5	Capsule	Bilberry (*V. myrtillus*) fruit extract, cellulose, vegetable capsule (modified cellulose), and magnesium stearate.
BL6	Capsule	Bilberry (*V. myrtillus*) fruit, gelatin, may contain one or more of the following‐ microcrystalline cellulose (plant fiber), magnesium stearate, and silica.
BL7	Capsule	Elderberry, bilberry extract, cellulose, gelatin (capsule), and silica. Main label states bilberry standardized.
BL8	Capsule	Bilberry (*V. myrtillus*) berry extract, blueberry (berry), gelatin capsule, and magnesium stearate.
BL9	Loose powder	Bilberry powder and freeze dried organic super concentrated.
BL10	Loose powder	Bilberry (*V. myrtillus*) powder and country of origin‐ Ecuador.
BL11	Loose powder	Bilberry extract powder, organic, freeze dried 4:1 extract (4× stronger), no fillers, and no chemicals or preservatives.
BL12	Liquid extract	Bilberry, vegetable glycerin, purified water, and alcohol free liquid extract.
BL13	Capsule	Bilberry (*V. myrtillus*) concentrated extract, maltodextrin, gelatin, and vegetable magnesium stearate.
BL14	Capsule	Bilberry and 100% natural.
BL15	Capsule	Bilberry extract.
BB1	Loose powder	Organic blueberry powder.
BB2	Dried fruit	Dried blueberries, no sugar added, and gluten, dairy, and allergen free.
BB3	Capsule	Blueberry (*V. corymbosum*), rice flour, gelatin capsule (gelatin, purified water), and silica magnesium stearate (vegetable grade).
BB4	Loose powder	Blueberry powder and silica.
BB5	Loose powder	Blueberry extract powder, organic, no fillers, and no chemicals or preservatives.
BB6	Liquid extract	Blueberry liquid, propriety fruit blend (agave concentrate, pomegranate concentrate, blueberry concentrate, cranberry concentrate, elderberry concentrate, green tea polyphenols (50%), liquid ionic minerals, purified water, natural flavors, citric acid, and potassium sorbate.
BB7	Liquid extract	Blueberry whole fruit extract, *V. corymbosum*, vegetable, glycerin, and purified water.
BB8	Capsule	Wild‐crafted blueberry complex (Alaska blueberry *V. alaskaense* How.), oval‐leaf blueberry (*V. ovalifolium*), alpine blueberry (*V. uliginosum* L.), dwarf bilberry (*V. cespitosum* Michx.) (fruit leaves, stems), vegetable cellulose (capsule), rice flour, maltodextrin, vegetable stearate, and silica.

### Reagents, chemicals, and standards

All chemicals, reagents, and standards used in this study were analytical or high‐performance liquid chromatography (HPLC) grade from Sigma‐Aldrich Chemical Co. (St. Louis, MO). Cyanidin‐3‐glucoside was purchased from Polyphenols Laboratories AS (Sandnes, Norway).

### High‐performance liquid chromatography condition for individual anthocyanin separation

High‐performance liquid chromatography ([HPLC]/DAD) (diode array detector) was used for anthocyanin separation, and mobile phase composition, gradient, flow rate, etc., were as described in Lee and Finn (2007), with the exception being a longer analytical column (Synergi Hydro‐RP 80Å, 250 mm × 2 mm, 4 *μ*m; Phenomenex, Inc., Torrance, CA), with a guard column (of the same phase) at the inlet of the analytical column. An Agilent 1100 HPLC (Agilent Technologies Inc., Palo Alto, CA) was used for this investigation. Elution peaks were monitored at 520 nm and 280 nm. Anthocyanin peaks were identified based on retention time, UV‐visible spectra, external standards (when available), verified authentic fruit with known anthocyanin profiles, and prior published research (Dossett et al. 2008; Finn et al. 2014; Hong and Wrolstad 1990a,b; Lee 2013, 2014, 2015; Lee and Finn 2007, 2012; Lee et al. 2012; anthocyanin profiles of small fruit samples that had been authenticated by plant taxonomist). Injection volume ranged from 3 to 10 *μ*L. Anthocyanins were expressed as cyanidin‐3‐glucoside. Samples were expressed in mg/100 g and mg per capsule, tablet, or teaspoon (abbreviated as tsp; assumed 5 g).

## Results and Discussion

Over 30% of the supplements (14 of 45) did not contain anthocyanins sourced from the claimed ingredients. Anthocyanin content for all 45 samples ranged from none detected (CB19, BL9, BL11, BL12, BB6, and BB7; *n* = 6) to 10,704.7 (BL8) mg/100 g, or 38.22 (BL8) mg per capsule, tablet, or tsp. basis. For the samples that were confirmed (*n* = 27) to originate from the labeled fruit, anthocyanins ranged from 3.4 (CB1) to 3513.2 mg/100 g (BL5) (see Table 2) or 0.04 (CB1) to 14.37 (CB16) mg/capsule, tablet, or tsp. (see Table 3). Unlike past analyses of liquid supplements (Gardana et al. 2014; Lee 2014), none of the liquid samples (CB19, BL12, BB6, and BB7) tested here contained any detectable anthocyanins. The supplements obtained for this study showed no preference for any particular form of fruit sourced to manufacture supplements. For example, cranberry supplements' labeled source materials included fruit, extract, concentrate, juice, and forms not stated. The form of source material was not useable as a prediction of the actual anthocyanin content contained within the supplement. The image in Figure 1 (cranberry supplements) shows the variety in appearances among the dietary supplements.

**Table 2:** Anthocyanin content (mg/100 g) of *Vaccinium* fruit dietary supplements (*n* = 45), ranging from none detected to 10,704.7 (BL8, mixed berry product and excluded from below). Only samples found to contain the *Vaccinium* fruit listed on package labeling (see Table 1) and were clearly distinguishable by high‐performance liquid chromatography (HPLC) data are presented here (*n* = 27)

*Vaccinium* fruit	Total samples evaluated	Number of samples not included in quantification^a^	Number of nonadulterated^b^ samples quantified	Mean (standard error)	Minimum ACY^a^	Maximum ACY
Cranberry	20	3	17	125.6 (43.1)	3.4	720.7
Lingonberry	2	2	0	b	b	b
Bilberry	15	10	5	1944.2 (553.7)	734.8	3,513.2
Blueberry	8	3	5	137.9 (51.1)	32.8	283.5

a Not included in the quantification data here due to absence of anthocyanin, too degraded, suspect profiles, or contained additional fruit (two bilberry samples and one blueberry sample) ingredients.

b Not determined.

**Table 3:** Anthocyanin content expressed as mg per capsule, tablet, or teaspoon (5 g) ranging from none detected to 38.22 mg (BL8). Only samples found to contain the *Vaccinium* fruit listed on package labeling (see Table 1) and were clearly distinguishable by high‐performance liquid chromatography (HPLC) data are presented here (*n* = 27)

*Vaccinium* fruit	Total samples evaluated	Number of adulterated and not included in quantification^a^	Number of nonadulterated samples quantified	Mean (standard error)	Minimum ACY^a^	Maximum ACY
Cranberry	20	3	17	2.47 (0.63)	0.04	14.37
Lingonberry	2	2	0	b	b	b
Bilberry	15	10	5	5.23 (1.22)	2.15	8.43
Blueberry	8	3	5	1.41 (0.63)	0.60	2.23

a Not included in the quantification data here due to absence of anthocyanin, too degraded, suspect profiles, or contained additional fruit (two bilberry samples and one blueberry sample) ingredients.

b Not determined.

### Supplements labeled to contain cranberry

**Figure 2:**
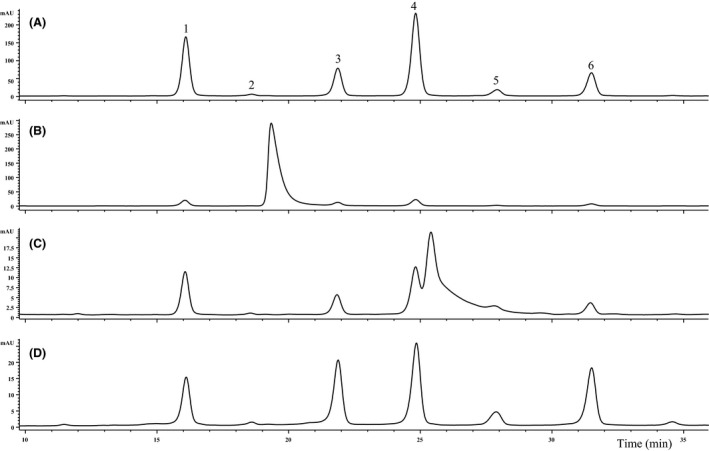
Cranberry and suspicious lingonberry dietary supplements anthocyanin profiles. Trace A (CB14) represents an authentic cranberry anthocyanin profile (as found in Lee 2013). The rest (B–D) are to demonstrate the difference in anthocyanin profile of other cranberry supplement samples, either containing artificial colorant (B, CB18), low anthocyanins with beet juice (C, CB20), or species adulterated lingonberry dietary supplement (D, LB2; presumably cranberry based on anthocyanin profile authentic lingonberry only contain the first three eluting peaks with peak 1 as the dominant; see Lee and Finn 2012). Peak assignments are 1 – cyanidin‐3‐galactoside, 2 – cyanidin‐3‐glucoside, 3 – cyanidin‐3‐arabinoside, 4 – peonidin‐3‐galactoside, 5 – peonidin‐3‐glucoside, and 6 – peonidin‐3‐arabinoside.

Seventeen (CB1‐CB17) of 20 cranberry supplement samples contained cranberry anthocyanins. A representative cranberry anthocyanin profile can be seen in Figure 2A (CB14). Cranberry products typically contain six anthocyanins; galactoside, glucoside, and arabinoside of cyanidin and peonidin (Lee 2013). Three samples (CB18, CB19, and CB20) did not have clear cranberry anthocyanin profiles, and are shown in Figure 2B (CB18) and Figure 2C (CB20). CB18 had FD&C Red Number 40 and FD&C Blue Number 2 listed in the ingredients, which may account for the peak eluting around 19 min (Fig. 2B). CB20 contained beet juice according to the label ingredient listing, and was found to be low in anthocyanins with extra peaks not characteristic of cranberries (see Fig. 2C). CB19 (the only cranberry liquid form obtained) contained no detectable anthocyanins, though cranberry concentrate was listed as an ingredient.

On a mg/100 g basis, cranberry supplements' anthocyanin ranged from 3.4 (CB1, tablet form) to 720.7 (CB17, capsule) (Table 2), or in a per capsule, tablet, and tsp basis ranged from no anthocyanin detected to 14.37 mg (CB16; powder form). The samples that contained cranberry anthocyanins, listed in increasing order of concentration (mg anthocyanin per capsule, tablet, or tsp basis in parentheses) were: CB1 (0.04) < CB2 (0.07) < CB6 (0.16) < CB3 and CB7 (0.17) < CB9 (0.23) < CB4 (0.35) < CB11 (0.38) < CB12 (0.49) < CB14 (0.85) < CB13 (1.25) < CB5 (1.34) < CB17 (1.51) < CB8 (2.57) < CB10 (3.70) < CB15 (14.29) < CB16 (14.37). Cranberry fruit has been reported to contain 25.7 to 92.1 mg/100 g in fresh weight (Vorsa et al. 2003). Although 100 g of four supplements (CB1, CB2, CB3, and CB4; all in dry‐form) contained less anthocyanin than 100 g of fresh cranberries, despite the significantly greater amount of fresh fruit needed to make 100 g of dehydrated cranberry powder.

### Supplements labeled to contain lingonberry

Only two (LB1 and LB2) lingonberry dietary supplements (both capsule form) were available at the time purchase. LB1 and LB2 samples were both labeled to contain *V. vitis‐idaea*. The anthocyanins of LB1 were too degraded to determine if it contained lingonberry, but it appeared to have a strong peonidin‐3‐arabinoside peak, which is not a main lingonberry (nor cranberry) anthocyanin (Lee and Finn 2012). Based on this, LB1 was excluded from the quantification summary tables. LB2 (see Fig. 2D) was a case of species adulteration as it contained cranberry, not lingonberry based on its anthocyanin profile. Authentic lingonberry anthocyanin profile can be found in Lee and Finn (2012), and only comprises cyanidin‐3‐galactoside (its primary anthocyanin), cyanidin‐3‐glucoside, and cyanidin‐3‐arabinoside. LB2 contained high levels of cyanidin‐3‐arabinoside (see Fig. 2D), peonidin‐3‐galactoside, peonidin‐3‐glucoside, and peonidin‐3‐arabinoside. Fresh lingonberry fruit anthocyanin concentrations have been found to ranged from 17 to 174 mg/100 g in fresh weight (Debnath and Sion 2009; Lee and Finn 2012).

### Supplements labeled to contain bilberry

**Figure 3:**
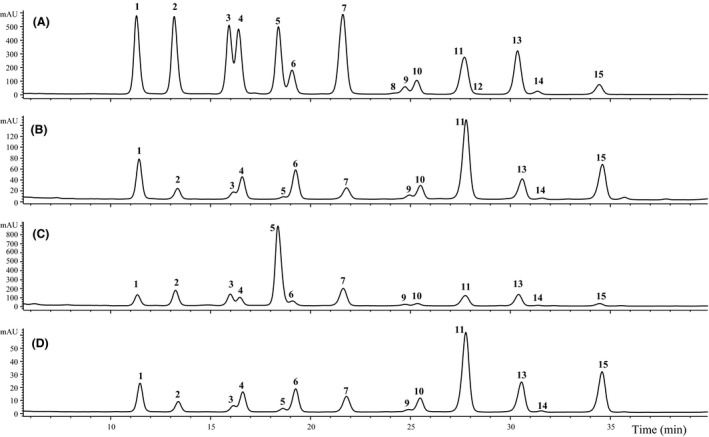
Bilberry (A, BL1), blueberry (B, BB2), mixed berries (C, BB8), and suspicious bilberry (D, BL6) dietary supplement anthocyanin profiles. BL1 (A) and BL2 (B) samples presumed authentic based on anthocyanin profile. Trace C (BB8) was difficult to determine authenticity from anthocyanin profile, since it was a mixture of four blueberry species (reported) and two addition blueberry ingredients (species unspecified). Trace D (BL6) was probably blueberry, not bilberry, based on its anthocyanin profile (compare the peak areas to B; see body of manuscript for more details). Peak assignments are 1 – delphinidin‐3‐galactoside, 2 – delphinidin‐3‐glucoside, 3 – cyanidin‐3‐galactoside, 4 – delphinidin‐3‐arabinoside, 5 – cyanidin‐3‐glucoside, 6 – petunidin‐3‐galactoside, 7 – cyanidin‐3‐arabinoside, 8 – petunidin‐3‐glucoside, 9 – peonidin‐3‐galactoside, 10 – petunidin‐3‐arabinoside, 11 – malvidin‐3‐galactoside, 12 – peonidin‐3‐glucoside, 13 – malvidin‐3‐glucoside, 14 – peonidin‐3‐arabinoside, and 15 – malvidin‐3‐arabinoside.

**Figure 4:**
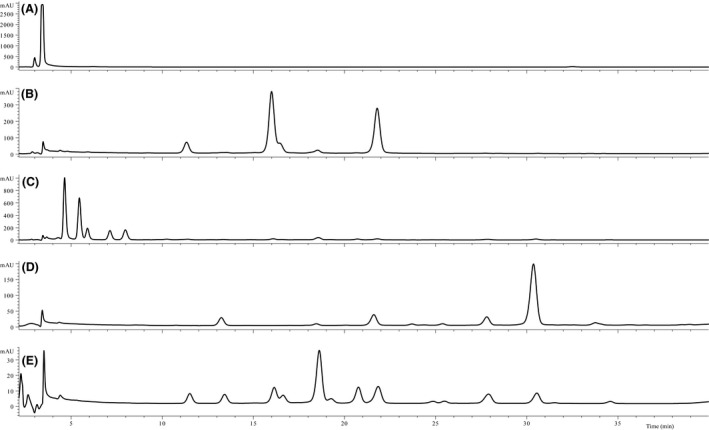
An array of adulterated bilberry dietary supplement samples anthocyanin profiles (BL9‐loose powder, BL10‐loose powder, BL14‐capsules, BL15‐capsules, and BL13‐capsules; represented in that order by traces A–E below). Bilberry sample BL6 is shown in Figure 3D. These anthocyanin profiles are presented as an example for future quality assurance assessments. Compared to bilberry anthocyanin profile in Figure 3A and Latti et al. 2008, it should be clear that these bilberry supplements are suspicious. For example, BL10's anthocyanin trace (Fig. 4B) is not *Vaccinium myrtillus* (bilberry), but that of *Vaccinium floribundum* (Andean blueberry; see Schreckinger et al. 2010 and Vasco et al. 2009).

The bilberry anthocyanin profile of BL1 is presented in Figure 3A. There were clearly 15 anthocyanins, as expected for bilberry (listed in Fig. 3), despite peonidin‐3‐glucoside (peak 8) sometimes being below detection threshold. Five bilberry (from BL1 to BL5) dietary supplement samples were quantified. The remaining bilberry samples (from BL6 to BL15) were found to contain no bilberry anthocyanins. One included unlisted ingredients (BL6) and two were purchased as bilberry mixed with other fruit (BL7 and BL8). BL6's anthocyanin profile showed it to be of blueberry origin, not bilberry (Fig. 3D; additional explanation in next section). BL7 was labeled (Table 1) to contain bilberry (80 mg) and elderberry (120 mg), but this could not be verified from anthocyanin profile alone. BL8 was labeled as a mixture of bilberry extract and blueberry fruit, but this too could not be determined by only the anthocyanin profile. The profiles of both BL7 and BL8 reveal bilberry anthocyanins; however, verifying the elderberry or blueberry portions would be impossible without knowing their mixture ratios and starting material anthocyanin profiles. BL10 represented another case of species adulteration, as it appeared to be sourced from *V. floribundum* Kunth (see Fig. 4B, for anthocyanin trace), also known as Andean blueberry or mortiño, and not *V. myrtillus* as labeled under ingredients. The anthocyanin profile of BL10 matched reports for authentic *V. floribundum* fruit (Vasco et al. 2009; Schreckinger et al. 2010), and contained delphinidin‐3‐galactoside, cyanidin‐3‐galactoside, delphinidin‐3‐arabinoside, cyanidin‐3‐glucoside, and cyanidin‐3‐arabinoside. BL10 also lacked the petunidin, peonidin, and malvidin containing anthocyanins of a true bilberry. Ecuador was the labeled country of origin for BL10 (Table 1).

Based on anthocyanin separation, the majority of the bilberry supplements contained unidentified material: examples shown in Figure 4A–E (BL9, BL14, BL15, and BL13, in the order presented). Three samples (BL9, BL11, and BL12) contained no anthocyanins. Both BL9 and BL11 were sold as packages of loose powder, appeared dark purple visually, and contained no anthocyanins. BL12, the only available bilberry supplement sold as an extract, was highly viscous and dark in color, but it too had no detectable anthocyanins. It should be noted that the anthocyanin profiles of bilberry fruit have not been shown to vary among geographic regions or with growing conditions (Latti et al. 2008; Gardana et al. 2014; Govindaraghavan 2014).

In mg/100 g basis, bilberry supplements' anthocyanin content ranged from 734.8 (BL1, capsule) to 3513.2 (BL5, capsule) (Table 2). Bilberry supplements' anthocyanins per capsule, tablet, and tsp. ranged from not detectable to 8.43 mg (BL5; capsule). Measurements of whole bilberry (*V. myrtillus*; whortleberry) fruit anthocyanins have ranged from 151 to 1310 mg/100 g in fresh weight (Latti et al. 2008; Gardana et al. 2014), or from 1971 to 3803 mg/100 g dry weight (Latti et al. 2008). Although comparisons of samples' to whole fruit anthocyanin content are problematic, as the supplements' labels suggested source materials as dehydrated extracts, concentrates, or whole fruit, but descriptions were not clear enough to make the distinction in most cases. The five samples that contained bilberry anthocyanins were (in increasing order; values in parentheses in mg per form sold): BL1 (2.15) < BL2 (3.48) < BL3 (4.33) < BL4 (7.75) < BL5 (8.43).

Since a consumer cannot currently tell visually if a bilberry supplement is actually made from bilberries, obtaining whole fruit forms (fresh, dried, or frozen) would be a safer source of bilberry phenolics. As previously mentioned, while samples BL9 and BL11 resembled dried dark berry powders, their chromatograms (BL9's shown in Figure 4A; BL11's chromatogram was similar) show that they clearly had no *Vaccinium* anthocyanins eluting (~3.4 min peak). Over 66% of the bilberry products purchased had inaccurate labeling. Bilberry product adulteration appears to be a global issue, with reports from Artaria et al. (2007), Gardana et al. (2014), Govindaraghavan (2014), and Penman et al. (2006), working with samples obtained in Italy, China (through an Australian distributer), and the U.S. marketplace. This work is the second study to find adulterated bilberry products in the United States.

### Supplements labeled to contain blueberry

A typical highbush blueberry anthocyanin profile is provided in Figure 3B (BB2) with individual anthocyanins are listed there as well. The same 15 anthocyanins found in bilberry are also found in blueberries, but petunidin‐3‐glucoside (peak 8) and peonidin‐3‐glucoside (peak 12) are not always quantifiable. Highbush blueberries contain a higher percent proportion of malvidin‐based anthocyanins than the delphinidin‐ and cyanidin‐based anthocyanins of bilberry (Lee et al. 2002; Latti et al. 2008). The blueberry supplement samples here had 52% malvidin‐, 23% delphinidin‐, and 12% cyanidin‐based anthocyanins, while authentic bilberry samples have been measured at 34% delphinidin‐, 34% cyanidin‐, and 18% malvidin‐based anthocyanin. As mentioned earlier, BL6 was a match for blueberry, not bilberry, at 57% malvidin‐, 18% delphinidin‐, and 9% cyanidin‐based anthocyanins (again, see Fig. 3D). Anthocyanin profiles can be used to distinguish the two species: *V. myrtillus* (bilberry) versus *V. corymbosum* (highbush blueberry) (see Fig. 3A. vs. Fig. 3B). Using anthocyanin profiles to distinguish different species has been previously demonstrated in other fruit (Lee 2013, 2014, 2015; Gardana et al. 2014), and is well established in aiding in chemotaxonomy (Hong and Wrolstad 1990a,b; Vorsa et al. 2003; Penman et al. 2006; Lee and Finn 2007, 2012; Latti et al. 2008; Lohachoompol et al. 2008; Debnath and Sion 2009; Vasco et al. 2009; Gardana et al. 2014; Lee et al. 2012; Finn et al. 2014; Dossett et al. 2008, 2011). A representative anthocyanin profile of rabbiteye (*V. ashei* Reade) blueberries can be found in Lohachoompol et al. (2008) demonstrating the differences in proportions of individual peaks from that of bilberry or highbush blueberry. Although processing actions (e.g., juicing, concentration, drying) frequently alter the anthocyanin proportions of final products (Lee et al. 2002; Schreckinger et al. 2010), there are cases where that does not occur (Mullen et al. 2002), and this should be kept in mind when comparing anthocyanin profiles.

Blueberry supplements BB1 thorough BB5 were quantified; BB6 and BB7 (both liquid extract form) were not included, as they contained no anthocyanins. BB8 was labeled to contain four different blueberry species (from the label – *V. alaskaense* How., *V. ovalifolium* Sm., *V. uliginosum* L., and *V. cespitosum* Michx.), a fruit, leaves, and stem complex, and two unspecified blueberry extracts. Without the availability of the relevant authentic anthocyanin profiles for comparison, as well as knowing the original prepared ratios, this mixture made it impossible to properly account for the individual ingredients (see Fig. 3C).

On a mg/100 g basis, blueberry supplements' anthocyanins ranged from 32.8 (BB1, powder) to 283.5 (BB5, powder) (Table 2). For the blueberry samples, the lowest and highest anthocyanins were both sold as loose powder. Blueberry supplements' anthocyanins on a per capsule, tablet, and tsp ranged from not detectable to 2.23 mg (BB2, dried fruit form). For the samples that contained blueberry anthocyanins (in increasing order; values in parentheses in mg per form sold): BB3 (0.60) < BB4 (1.17) < BB5 (1.42) < BB1 (1.64) < BB2 (2.23). Whole blueberry anthocyanins have been reported from 109 to 384 mg/100 g of fresh weight berries (Gao and Mazza 1994; Lee et al. 2002; Yousef et al. 2013).

For the quality assurance of dietary supplements, routine anthocyanin separation methods are needed, along with databases of these phenolic profiles from multiple laboratories using authenticated fruit samples. But conflicting data will still occur. For example, Lowenthal et al. (2013) made tentative peak assignments for some of the NIST (National Institute of Standards and Technology) *Vaccinium* fruit standards that could not be corroborated. They reported finding peonidin‐3,5‐diglucoside in American cranberry (Standard Reference Material 3281), when others have demonstrated that diglucoside‐containing anthocyanins are not present in typical American cranberry (see Fig. 2A) (Hong and Wrolstad 1990b; Lee 2013). Other cases of misidentification are shown in Lee et al. (2012). A deeper knowledge base of repeat findings for authentic fruit anthocyanin profiles is necessary, since availability of purified anthocyanin standards are limited; although more are available than for other phenolic classes, such as proanthocyanidins or ellagitannins (Lee et al. 2012; Lee 2013). Uncorroborated work (e.g., Lowenthal et al. 2013) only reinforces the need for developing accurate quality control assessments that are available to everyone.

Every analytical method has limitations. For example, AOAC method 2005.02 method (Lee et al. 2005) was validated to provide a simple method to determine anthocyanin concentration, it is an economical and simple method for quantification, but it is unable to distinguish among individual anthocyanins. It can quickly determine if a sample contains anthocyanin, as other red pigments (i.e., carmine, betalain, FD&C Red Number 40) will not undergo this color shift with pH change (Lee et al. 2005). A good qualitative anthocyanin separation method, via HPLC, will aid dietary supplement quality assessments, but again the need for a comprehensive fruit and vegetable anthocyanin database remains.

## Conclusion

From the results of this study, eating whole fruit for its nutritional value might be safer and more economical until there is greater assurance of dietary supplements' contents. The high percentage (>30%) of samples that did not contain anthocyanins from the fruit sources listed as ingredients underscore the need for quality control standards for dietary supplements sold in the United States. Improved labeling information would aid consumers in understanding the anthocyanin content, or amount of fruit, in a product. The low quality of some products available in the marketplace for this study was surprising. Of the samples that were confirmed to contain the *Vaccinium* species listed on the label, there were 212‐fold (cranberry), fivefold (bilberry), or ninefold (blueberry) differences between the lowest and highest anthocyanin content in their respective supplements. Anthocyanin profiles can be used as a quality and authenticity indicator, but once the product contains multiple fruits with unknown ratios, using anthocyanin profiles to determine authenticity is complicated. Although anthocyanin profiles can screen botanical ingredients and products when used in combination with other authentication techniques currently available (Cordella et al. 2002). The creation of an anthocyanin profile database could immediately help advance the quality of dietary supplements available to consumers, even if it only assisted processors in verifying their fruit ingredient sources.

## Conflict of Interest

None declared.
